# The Secret of Youth

**Published:** 1892-04-23

**Authors:** 


### The Doctor's Armchair.

### THE SECRET OF YOUTH.

A very natural object for the medical man to propose to himself is the long life and happiness of his patients and friends. It is taken for granted that all the doctor's patients are his friends, just as all the clergyman's parishioners are his. If it be objected that this is idealising too much, the answer is that if we do not idealise we fall to a pitifully low level of intellectual and general effort. Happiness is an indispensable condition of perfect health. The man who is not happy is not healthy. What is here meant by happiness is not necessarily pleasurable excitement, but an abiding condition of mind made up of several factors, two of the chief of which are a heartfelt contentment with one's lot and a reasonable hope of success in one's work. Such happiness as this the doctor must feel it incumbent upon him to promote in his patients, because, as we have said, it is an indispensable condition of health. Moreover, the absence of inward contentment and hopefulness is often due to ascertainable physical causes; and the presence of such a state of mind may frequently be restored and maintained by other ascertainable physical causes. But even if it were otherwise, there is surely no barrier interposed between the physical and the mental in the physician's work. On the contrary, whenever we see a case of a "mind diseased," we see also that it is generally the physician who is first called in to "minister" to it.

The "wear and tear" of life is one of the commonest expressions in everyday use. Such terms as "overstrain," "breakdown," "nervous depression," and "worry," tell their own tale. It is as proper to speak of the "breakdown" of a man as of the breakdown of a ship or a house. If the house be broken down, it is broken down in one way, and can only be repaired in one way. It is to be repaired mechanically and from without. But if a man be broken down he must be repaired in a different way; he must be built up, not from without but from within; not mechanically but chemically and vitally. This is a different and much more difficult work.

What is the innermost inwardness, the real centre and most secret point of radiation in a rational being like man? However philosophy may answer this question, every-day experience answers it in one way, and in one way only. The mind is the centre as well as the standard of the man. If the mind be experiencing well-being, then all is well with the man; at least so far as he himself is concerned. The converse also is true. If the mind be distraught and far from contentment, then nothing is well with the man. He cannot be made to admit that he is well; he cannot be made to feel that he is well; he is not well; he is sick; and he needs to be dealt with by a rational and adequate therapeutic.

Men who are old enough to weigh up the world in the scales of their judgment, and to decide whether they will resolutely exercise a conscious will in it, or merely drift away to the shore of death on the tide of circumstances, feel a constant necessity for some vitalising force which shall maintain, and, if possible, increase in them the energy of youth. Youth hopes all things, and dares all things. It has a splendid temper to confront the world with. This hopefulness and courage are worth more than gold or precious stones to their possessor. In early life hopefulness and courage have not experience to guide them; and so they often effect much less than they seem fitted to effect, to the very great disappointment of the young man or young woman, their possessor. But if the sober experience of middle-age could constantly animate itself with the hopefulness and daring of youth, there are few things which are possible to man which it might not accomplish. This is what the observant physician perceives to be the prime want of most of his middle-aged and older patients. They are fires that are burning low, volcanoes that are threatening to become extinct. Is it inevitable that they burn low? Is this the history of every man—a kindled fire, a blazing flame, dying embers, and then ashes, cold, and dead? No! It is not quite the history of every man. Here and there are men and women who are young even in old age.

If it is admitted that up to this point the language of sober truth, and not of effervescent enthusiasm, has been spoken, then perhaps readers who are anxious, like the writer, to push forward more deeply into the less obvious regions of fact and influence that affect mankind, will be willing to ask sympathetically what the secret is of a youthfulness that endures to the end of life, and that almost compels one to think it may endure even beyond life? Is there such a secret? Is there only one, or are there several? Can one, or all of those secrets be made available for universal use? Questions like these are apt to be considered unpractical. They are unpractical to the impatient and the untrained. But it is quite beyond dispute that a certain number of aged persons have all the appearance of perennial youth about them; and that they seem to be the only kind of men and women who find old age even so much as tolerable. So urgent are the manifold disabilities of declining years in most persons that any discovery in science which gives promises of abating them, even in a small degree, is hailed by an expectant world as little less than a miracle. When Brown-Sequard announced that he had produced an elixir which would restore the aged to youth, his name was in everybody's mouth, from China to Western America, and from the islands of the southern seas to the remotest regions of the ice-bound north.

The secret of enduring youth, if secret there be, is to be looked for within a man, not outside him. Science, in recent years, has so devoted itself to the objective that it has almost been tempted to question the very existence of the subjective. But the tide is turning. The whole history of bacteriology is a standing protest against the grosser pathologies and physiologies. It is as clear as noon-day to any man who has even an elementary knowledge of scientific facts and principles that if we desire to know what things are in themselves we must condescend to be patient, and to take counsel of the minute. We must not only go as far as the microscope will show us the way, but even further; and in the last resort it will often be found necessary to call in the aid of a psychology whose material nidus is quite impossible of demonstration. If the present writer were asked to say what, in his judgment, is the most available secret of enduring youth and youthfulness, he would answer: "The striving always for the best in motive and work." Work itself is a sovereign cure for manifold ills: the best work is the best cure: when the work and its motive are alike the greatest and the noblest, the inward man is in a condition of such perfect wholesomeness that the ills of life have hardly power to annoy, much less to damage. The well-being of man has always been the well-being of mind: the triumph of man has always been the triumph of mind. These are absolute, incontestable, and a thousand times demonstrated facts. At the close of an article it is impossible to enter upon a new and scientific proof, but whoever will take the trouble to think out our thesis for himself will not fail in the construction of a complete and satisfactory demonstration.

